# Using virtual worlds for role play simulation in child and adolescent psychiatry: an evaluation study

**DOI:** 10.1192/pb.bp.113.044396

**Published:** 2014-10

**Authors:** Aaron K. Vallance, Ashish Hemani, Victoria Fernandez, Daniel Livingstone, Kerri McCusker, Maria Toro-Troconis

**Affiliations:** 1 Imperial College London; 2 Springfield University Hospital, London; 3 University of the West of Scotland, Paisley; 4 University of Ulster, Derry

## Abstract

**Aims and method** To develop and evaluate a novel teaching session on clinical assessment using role play simulation. Teaching and research sessions occurred sequentially in computer laboratories. Ten medical students were divided into two online small-group teaching sessions. Students role-played as clinician avatars and the teacher played a suicidal adolescent avatar. Questionnaire and focus-group methodology evaluated participants’ attitudes to the learning experience. Quantitative data were analysed using SPSS, qualitative data through nominal-group and thematic analyses.

**Results** Participants reported improvements in psychiatric skills/knowledge, expressing less anxiety and more enjoyment than role-playing face to face. Data demonstrated a positive relationship between simulator fidelity and perceived utility. Some participants expressed concern about added value over other learning methods and non-verbal communication.

**Clinical implications** The study shows that virtual worlds can successfully host role play simulation, valued by students as a useful learning method. The potential for distance learning would allow delivery irrespective of geographical distance and boundaries.

Medical education has a long-established interest in using virtual reality simulators to train doctors.^1^ Such technology is now replicated on the internet in the form of virtual worlds; this software enables users to navigate ‘avatars’ through virtual environments and communicate with other avatars online. Although a number of medical educational and psychoeducational applications of virtual worlds have been developed,^2^ there remains a paucity of research.^3^ Moreover, like virtual reality simulators, current virtual world medical applications generally involve learners interacting with the virtual environment (e.g. using objects, accessing text or audio-visual media through embedded pop-up windows) rather than with people. Research shows however, that without the interpersonal interaction, the application struggles to capture the complexity of clinical interaction;^4-6^ Rampling *et al*^4^ conclude that conversational interaction between avatars would significantly improve the learning experience.

Our study aimed to harness interpersonal aspects of Second Life technology (a free-to-access online virtual world) to develop and evaluate a real-time clinical role play simulation. We are unaware of research evaluating Second Life for teaching child psychiatry, a specialty embracing interpersonal dynamics and complex decision-making.^7-9^ Simulation can standardise learning, particularly important given medical students’ variable exposure to child psychiatry.^10^ The specialty also involves risk management in patients vulnerable by virtue of age and mental disorder; simulation could provide a standardised learning environment to safely explore risk.^11^

Kneebone *et al* argue that simulation involves the ‘suspension of disbelief and then “buy-in” to the simulated experience’.^12^ Virtual worlds could enhance role play simulation fidelity, helping students suspend belief that they are interacting with a teacher, while engaging them deeper with the clinical narrative.^13^ Few medical education studies have previously explored the relationship between simulation fidelity and educational utility. This study asks whether medical students perceive a Second Life role play simulation as useful. Secondary questions explore: emotional aspects of learning, technology’s ease of use, whether the simulation delivers ‘good enough’ fidelity and its relationship to perceived utility.

## Method

The study, approved by Imperial College Research Ethics Committee, comprised part of a Jisc multisite project.^14^

### Participants

We recruited ten Imperial College penultimate-year medical undergraduates post-psychiatry or paediatric attachments. Sample size matched the methodologies of small-group teaching and qualitative research. We advertised internally and emailed information sheets to responding students, accepting participants consecutively. The research session immediately followed the teaching session. The session began with participants completing consent forms, receiving role play tasks and 30 minutes’ training on Second Life. Participants received £45 retail vouchers to reimburse time.

### Teaching session

**Fig 1:**
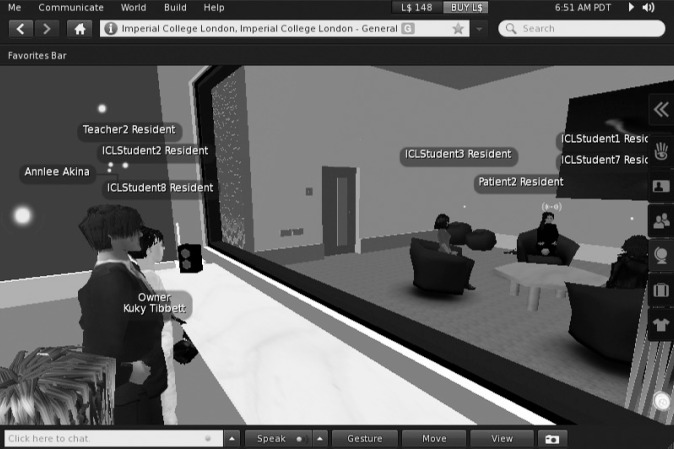
The role play session in the virtual clinic. The teacher plays an adolescent avatar (avatar on right-hand side, facing forwards, with an overhead icon). Role-playing participants are in the clinic room; observers are watching through the ‘virtual’ one-way screen.

Two consecutive 90-minute teaching sessions were conducted in computer labs, each with a randomly assigned group of five participants. The teacher operated from a different lab. All teaching occurred on Second Life Viewer (version v.3.3.4), within a virtual prefabricated clinic designed from a real clinic for authenticity (Fig.1; colour version given in online Fig. DS1).^12^ Users only interacted through their avatars, communicating via audio-microphone headsets (technical details available from the authors on request).

Learning outcomes covered psychiatric assessment and management, communication and professional skills. The session was divided into briefing, role play, reflective and debriefing stages (Table 1).^15-17^ The role play involved students using their avatars to role-play with the teacher playing a ‘depressed teenager’ avatar.

### Research methods

Research was primarily qualitative, since the goal was to explore in-depth attitudes towards a novel application of learning technology which had hitherto been minimally researched. Furthermore, the teaching was small-group by nature, which particularly lent itself to qualitative research. We included an additional quantitative method to evaluate the degree of consensus and ambivalence across the group relating to the various research questions. The mixed method enables triangulation of data interpretation.

### Questionnaire

A literature search showed no evidence of an accepted standard validated questionnaire on clinical simulation. We developed and piloted an internet-based questionnaire encompassing Likert-style questions and open-box comments, in accordance with our research questions and respective theoretical foundation (online Table DS1).^15-18^ We incorporated a questionnaire from Bonanno & Kommers,^19^ chosen for its relevance but adapted to make explicit reference to Second Life rather than ‘computer gaming’ more generically. Additional questions explored usability, utility and prior experience.^15,20^ Analyses of demographic variable influence and fidelity-utility relationship were undertaken with SPSS Version 18, Windows XP, although the small sample size was expected to lack power for statistical significance. Open-box data fed into the thematic analysis.

**Table 1:** The stages of the 90-minute teaching session

Teaching session stage	Process	Educational models^15^^,^^17^
Briefing session	As ‘teacher avatar’, teacher reviews the principles of child psychiatric assessment, depression and self-harm; participants adopt ‘clinician avatars’.	Discursive dialogue enables the teacher to elicit and consolidate students’ prior understanding. Students enter learning armed with a biography of experience, expectation and knowledge.
		
Role play	The ‘teacher avatar’ leaves the virtual clinic; the teacher returns as a ‘patient avatar’ presenting ‘post-overdose’. The teacher acts flexibly, following a narrative crib sheet. Three participants per group respectively assess: presenting complaint, mental state and psychosocial history. The two remaining participants retire to an adjacent ‘virtual’ observation room, observing the role play through a ‘virtual’ one-way screen.	The role play represents an episodic learning experience with cognitive, physical, attitudinal, emotive and sensory components. Learners receive real-time intrinsic feedback through the responses of the ‘patient avatar’, enabling them to experiment with different strategies and learn from others’ responses.
		
Student reflection	Students write reflective notes on an electronic form, accessed from an icon embedded in the Second Life environment. Using SLOODLE software (www.sloodle.org), these forms are then submitted electronically to the teacher.	Written reflection aims to facilitate learning through appraisal of one’s experience and trialling in future situations.
		
Debriefing session	The tutor facilitates a reflective discussion, guided by students’ submitted reflections, and delivers general and individual feedback.	Learning is further facilitated through the teacher’s extrinsic feedback.

### Nominal group and thematic analysis

Compared with conventional focus groups, nominal groups minimise individual dominance and encourage theme breadth.^21^ The process began with participants writing their own responses to the question: ‘What are the advantages and the disadvantages of learning in Second Life (compared with other methods)?’ Views were then shared one by one in round-robin fashion and recorded and coded on a whiteboard. A discussion clarified and rationalised single points into larger or smaller themes where appropriate. Each participant then anonymously ranked their top ten themes. Each rank related to a score (first place 10 points, second place 9 points, etc.), which enabled generation of an overall group ranking based on collective scores. Further group discussion allowed final reordering of the top ten themes according to consensus.

A.K.V. moderated, recorded, transcribed and thematically analysed the nominal group. Themes generated by the nominal group guided the first round of coding in the thematic analysis, whereas further rounds of analysis interrogated the data. We are unaware of this approach being used in other research studies, but applied it to achieve a balance between the nominal group procdures democracy and breadth of participant-derived themes with a more in-depth thematic analysis. Themes were explicitly identified and analysed by a realist perspective.^22^

## Results

Participants were aged 22-24 years; six were female, four were male. Seven had heard of Second Life, but none had experience of it; all regularly played computer games; nine had role play experience.

### Evaluation data from the questionnaire

Quantitative data from the questionnaire are shown in Table DS1; statements in the adapted Bonanno & Kommers’ questionnaire^19^ are grouped into four domains: ‘affective’, ‘behaviour’, ‘perceived control’ and ‘perceived usefulness’. As per the original study, adapted Bonanno & Kommers’ statements were each scored on a 1-5 scale, with positive attitudes scoring higher. Statement scores are combined to provide domain scores and a composite ‘general attitude’ score. These scores were evaluated for differences with respect to gender, ethnicity, prior gaming use and experience of using role play in training. The only analysis meeting statistical significance was that more males (*v*. females) and those with experience in online multiplayer games (*v*. those with no experience) agreed that ‘I can make the computer do what I want it to do while learning using Second Life’ (both *P*=0.048, two-tailed Fisher’s exact test). This result needs to be interpreted cautiously given the multiple statistical analyses performed.

To explore the relationship between simulation fidelity and perceived usefulness, a specific analysis compared participants who agreed that ‘the graphics/visuals were sufficient to make the role play appear realistic enough’ with those disagreeing or uncertain (Table 2). Those demonstrating a positive attitude about simulation fidelity scored significantly higher across all four domain scores and on the total ‘general attitude’ score. Data consensus within this section supports the likelihood that the significance is genuine.

### Nominal group data

Delays following unexpected software maintenance resulted in one participant having to leave before the nominal group started; two more left before voting. Twenty-six themes were identified and ranked by their total score (Table 3). Thematic analysis results are covered in the next section.

## Discussion

We developed a novel teaching session using Second Life as a role play simulation to teach child psychiatric assessment to medical students, and evaluated it with a multimodal research approach. This section discusses results, structured according to thematic analysis’ domains. The key quotes are reported in online Table DS2.

### Utility of the Second Life role play to facilitate learning

Whereas e-learning frequently involves knowledge transmission, participants valued the Second Life simulation for also developing skills (Table DS1); furthermore, all participants thought that making/observing mistakes helps avoid making them with real patients.^11^

Other advantages identified include ‘standardising clinical experiences’, voted for by 3/7 nominal group participants, with two more referencing it in the group discussion. The group also identified a closely related theme: that Second Life simulation can expand clinical exposure, also reported in Toro-Troconis *et al*.^6^ Although standardisation could be attained by any role play activity, participants noted the technology’s capacity to mimic illnesses, for instance colourful clothes in mania or thinness in anorexia.

Combining several nominal group themes revealed that participants valued virtual worlds’ potential to allow acclimatisation to clinical environments before interacting with real patients, replicating other studies.^4,6^ Acclimatisation also encompasses anxiety reduction. One participant argued that simulation may, however, lull students into a ‘virtual comfort world’, preventing graduation to real clinical environments.

As in other studies,^4,6^ our participants identified ‘distance learning’ as an added value of Second Life; the nominal group ranked it third. Moreover, 5/7 participants voted for a closely related issue: Second Life can save time, effort and money on commuting or venue-booking. Furthermore, it may allow learning by anyone at any place, even beyond a single institution. It could therefore be applied to help doctors learn in countries where specific services (e.g. child psychiatry) or clinicians are limited.

### Limitations of the Second Life role play

Like other learning technology studies,^23^ participants saw the Second Life simulation as a supplement, rather than substitute, for methods such as patient interaction: ‘nothing replaces the “real thing”’. Discourse relating to this theme focused on the general application of the simulation. In contrast, when specifically discussing its application for child psychiatry, participants instead valued the potential to expand their clinical experience to situations they would not normally encounter. Indeed, the simulation was partly conceived to compensate for the limited and variable exposure to child psychiatry across medical school curricula.^10^ Although access to real-life scenarios would arguably be the ideal learning experience, in practice curricular time pressures and ethical issues challenge such access.

**Table 2:** Comparing participants’ attitudes of fidelity with other attitude domain scores

	Median scores (IQR)^a^		
Domain scores	Uncertain/disagree/strongly disagree	Agree/strongly agree	*P*^b^
Usefulness	13.50 (10.75-14.50)	16.50 (15.25-19.25)	0.02
			
Behaviour	11.50 (8.75-13.25)	14.50 (14.00-18.00)	0.02
			
Affective	20.00 (15.75-23.50)	25.50 (24.00-27.00)	0.03
			
Control	17.50 (16.00-19.00)	24.50 (21.50-26.75)	0.01
			
General attitude	62.00 (55.75-68.50)	80.00 (76.25-90.50)	0.01

IQR, interquartile range.

^a.^ ‘The graphics/visuals were sufficient to make the role play appear realistic enough’.

b. In comparison between the two groups, using two-tailed Mann-Whitney statistical analysis.

The sixth-ranked nominal group theme comprised the limited ‘added value’ compared with real-life role play or other learning opportunities; some participants noted that standardisation and acclimatisation could be delivered by real-life or actor role plays. The questionnaire meanwhile revealed discrepant data (Table DS1). On one hand, half the participants agreed they learned nothing more than what is achievable by real-life role play and most agreed that most things learned from role-playing in Second Life is obtainable through other means. In contrast, only two participants would avoid learning using Second Life, whereas only two disagreed that using Second Life justified the effort.

### Aspects of simulation

Simulation involves detaching from real life and absorbing oneself in the simulated scenario.^16^ Virtual worlds can engage as simulations: in Slater *et al*’s 21st-century reprise on ‘Milgram’s obedience experiments’,^13^ participants scored highly on subjective and objective measures of stress on seeing an avatar ‘electrocuted’.

The relationship between simulation fidelity and educational utility has rarely been explored in medical education research. In our study, by detaching students from the teacher and by asking them to interact with an adolescent-appearing avatar, we hypothesised that fidelity may be enhanced. Results showed that some participants felt that being detached from the teacher improved fidelity; however, most felt that fidelity was compromised by the limited expression of body language.

Some participants also noted difficulty in concentrating, suggesting several factors: the need to occasionally multitask, overhearing participants in the real world, and trying to master the technology.

**Table 3:** ‘Top ten’ themes - positive (coloured) and negative - generated, voted, ranked and ordered by the nominal group

Rank	Themes generated	Domain^a^	Total score	Participants rating theme in ‘top ten’ *n*
1	Lack of avatar realism, especially limited/stereotyped body language, impeded psychiatric assessment and group interaction, both of which depend on non-verbal communication	Sim	57	7
				
2	Conducting the role play in Second Life reduced stress/worry, which eased engagement	Affect	35	5
				
3	Second Life could facilitate distance learning - students and (expert) teachers can access it from different geographical locations	Utility	32	4
				
4	Audio delay on Second Life resulted in participants talking over each other	Op	23	4
				
4	Second Life increases potential to act disinhibited (e.g. changing avatar’s appearance, walking around), which distracts from learning	Sim	23	4
				
6	Role-playing on Second Life does not add value compared with existing learning opportunities	Limitation	19	2
				
7	Second Life provided a useful conceptual barrier between teacher and student, which facilitated role play but still allowed teacher to effectively highlight clinical aspects	Sim	24	6
				
8	Second Life would allow teaching to be flexible in terms of space (allows for a private/convenient location; does not require booking rooms) and time (can be done at any hour)	Utility	21	5
				
9	Role-playing in Second Life was interactive and more fun than role-playing in real life	Affect	21	4
				
10	Compared with interacting in real life, it was harder to maintain concentration throughout the session on Second Life (e.g. as less varied non-verbal communication)	Sim	16	3
				
10	Conducting the session in the same physical room meant you could hear people in both real life and in Second Life; the audio delay was distracting	Sim	16	2

Op, operational issues; Sim, aspects of simulation.

a. Domains were defined retrospectively by the subsequent thematic analysis.

### Detachment from the ‘real’

The seventh-ranked nominal group theme defined Second Life as a useful ‘conceptual barrier’, weakening the sense of interacting with a teacher; 6/7 participants voted for it. However, questionnaire data revealed that most still struggled to ‘switch off’ from thinking the patient was actually the teacher. Furthermore, no role players found that Second Life helped get into role compared with real-life role-playing, implying that any advantage derived from the conceptual barrier may be offset by the simulation’s low-fidelity body language. Intriguingly, the fourth-ranked nominal group theme identified the ‘conceptual barrier’ as leading to disinhibited behaviour. Perhaps virtual reality leads users to behave to different norms, particularly as they explore its limits and rules.

### Avatars’ low-fidelity expression of body language

Questionnaire data showed that most participants expressed difficulty in feeling connected with the patient. The simulation’s low-fidelity expression of body language was probably a primary contributing factor: the nominal group identified and ranked this theme highest, and related this to hindering patient engagement and group interaction. Many noted the importance of non-verbal communication to psychiatric practice, and this is reflected in empirical research showing how psychiatric disorders may influence expression of non-verbal communication.^24^

Factors contributing to the low-fidelity body language include limited graphics, although questionnaire data showed variation as to whether this seriously compromised fidelity: four participants thought graphics were sufficient to make the role play appear realistic enough, whereas four disagreed. Another factor is the teacher’s inexperience with Second Life; observers noted that gestures occurred only sporadically. Greater teacher experience may have improved fidelity: half the participants agreed that realism would be enhanced with more gestures.

Questionnaire data suggest a positive relationship between simulation fidelity and perceived utility. First, six participants agreed that if the simulation appeared exactly like real life, then the session would have been significantly more useful. Second, participants who agreed that ‘the graphics/visuals were sufficient to make the role play appear realistic enough’ had significantly higher computed scores on all four Bonanno & Kommers’domains, including ‘perceived usefulness’ (Table 2).^19^

Given that participants rated an improvement in knowledge and skills, by implication the audio-visuals were arguably at least ‘good enough’. However, some participants experienced the limited representation of body language as significantly detracting from utility, if not compromising it altogether. Surgical simulation research shows examples where low-fidelity simulators still compete favourably with high-fidelity counterparts.^25^

Some participants reported that low-fidelity body language impeded group interaction. Nevertheless, a reasonable flow of dialogue was observed.

### Affect

Various educational models emphasise emotional aspects to learning.^15,16,18^ Indeed, the nominal group identified two distinct ‘top ten’ themes relating to positive affect, although the thematic analysis revealed two participants reporting stress or boredom, both relating to the limited visual cues.

Whereas interacting with patients and role-playing can unsettle students,^9,26^ the second-highest nominal group theme indicated that Second Life attenuated anxiety, thus improving role play engagement. The simulation’s conceptual barrier may have reduced anxiety by facilitating detachment from the teacher. Several participants also described feeling less anxious compared with other small-group settings, feeling more confident asking questions or voicing thoughts. The relationship between anxiety and performance is, however, more equivocal: four participants agreed that being more relaxed led to better performance, whereas three disagreed. Some participants noted that anxiety may enhance performance.

The ninth-ranked nominal group theme was that role-playing in Second Life was interactive and more fun than role-playing in real life. The thematic analysis identified six participants describing the session as ‘interesting’ or ‘fun’.

Overall, questionnaire data support the qualitative data (Table DS1). Moreover, the ‘affect domain’ score was high, exceeding comparative scores in a solo game-based Second Life application.^27^ Perhaps enjoyment was enhanced by group interaction.

Many participants noted the novelty of the teaching, but would students enjoy it long term? Lee *et al*^18^ showed that students’ perceived enjoyment of a novel internet-based learning medium was significantly associated with future intention.

### Operational and practical issues

Despite this being their first experience of Second Life, and with only 30 minutes’ training, difficulties navigating were not raised in the nominal group; questionnaire data showed that most participants felt comfortable with the technology, with some exceptions. This contrasts with the difficulty experienced by students in Rampling *et al*’s Second Life study^4^ who received only online guidance. This may reflect the value of face-to-face training, however brief. Although all participants play computer games for leisure, some reported that older, ‘digitally naive’ teachers may have more difficulty.^28^

Although fractional, audio delay (the fourth-ranked nominal group theme) was experienced as a challenge to group interaction, particularly as participants could also overhear each other in the ‘real world’ room.

Finally, the Second Life role play requires logistics (e.g. setting up log-on times) and initial resources (e.g. computers, headsets). Although designing the virtual child and adolescent mental health service required initial investment, interested readers are welcome to use it (register with Second Life, then visit http://slurl.com/secondlife/Imperial%20College%20London/150/86/27/).

Participants identified ways that Second Life could actually represent cost-effective learning, relating to the benefits of distance learning or saving on actors.

### Study limitations

Recruitment bias may have occurred if particularly enthusiastic students (for psychiatry, technology or education) applied. Furthermore, students at Imperial College - a predominantly science- and technology-oriented institution - may differ to those elsewhere, in that they may be interested particularly in science and technology and thus more likely to be enthusiastic towards innovative learning technology.

Although the nominal group process helps generate breadth of data, the structured approach may restrict in-depth exploration of themes. Admittedly, the thematic analysis aimed to compensate for this, but dialogue still proceeded in accordance with the nominal group process. Thematic analysis is open to subjective interpretation, particularly as only one researcher was involved.

The teacher had limited experience of Second Life; participants’ concerns about body-language expression may therefore reflect the teacher’s lack of utilisation rather than an intrinsic software issue.

It is important to emphasise that this study evaluated the student experience; although participants generally perceived an improvement in their skills and knowledge, this does not necessarily translate into objective changes in academic performance.

Finally, although some questions explicitly incorporated a comparison (e.g. to real-life role-playing), most were open. Likewise, the nominal group question was open-ended. Students may have made implicit responses with a comparative teaching method in mind. A future study may benefit by making comparisons more explicit or by directly incorporating a control group (e.g. real-life role-playing). Another direction could involve more in-depth qualitative analysis.

## References

[1] Merril JR Notaroberto NF Laby DM Rabinowitz AM Piemme TE The Ophthalmic Retrobulbar Injection Simulator (ORIS): an application of virtual reality to medical education. Proceed Ann Symposium Computer Application Med Care 1993; 16: 702–06 PMC2248014 1482962

[2] Gorini A Gaggioli A Vigna C Riva G A second life for eHealth: prospects for the use of 3-D virtual worlds in clinical psychology. J Med Internet Res 2008; 10: e21 18678557 10.2196/jmir.1029 PMC2553247

[3] Peachey A Gillen J Livingstone D Smith-Robbins S Researching Learning in Virtual Worlds (1st edn). Springer, 2010

[4] Rampling J O’Brien A Hindhaugh K Woodham L Kavia S Use of an online virtual environment in psychiatric problem-based learning. Psychiatrist 2012; 36: 391–6

[5] Toro-Troconis M Mellström U Partridge M Meeran K Barrett M Higham J Designing game-based learning activities for virtual patients in Second Life. J Cyber Therapy Rehabil 2008; 1: 227–39

[6] Toro-Troconis M Roberts N Smith S Partridge M Zagalo N Students’ perceptions about delivery of game-based learning for virtual patients in Second Life. In Virtual Worlds and Metaverse Platforms: New Communication and Identity Paradigms (eds Zagalo N Morgado L Boa-Ventura A): 138–48 IGI Global, 2011

[7] Dave S Simulation in psychiatric teaching. Adv Psychiatr Treat 2012; 18: 292–8

[8] Holsbrink Engels G Using a computer learning environment for initial training in dealing with social-communicative problems. Br J Educ Technology 2001; 32: 53–67

[9] Nestel D Tierney T Role-play for medical students learning about communication: guidelines for maximising benefits. BMC Med Educ 2007; 7: 3 17335561 10.1186/1472-6920-7-3 PMC1828731

[10] Sawyer M Giesen F Walter G Child psychiatry curricula in undergraduate medical education. J Am Acad Child Adolesc Psychiatry 2008; 47: 139–47 18176334 10.1097/chi.0b013e31815cd9e0

[11] Ziv A Wolpe PR Small SD Glick S Simulation-based medical education: an ethical imperative. Simulation Healthcare 2006; 1: 252–6 10.1097/01.SIH.0000242724.08501.63 19088599

[12] Kneebone R Nestel D Wetzel C Black S Jacklin R Aggarwal R The human face of simulation: patient-focused simulation training. Acad Med 2006; 81: 919–24 16985358 10.1097/01.ACM.0000238323.73623.c2

[13] Slater M Antley A Davison A Swapp D Guger C Barker C A virtual reprise of the Stanley Milgram obedience experiments. PLoS One 2006; 1: e39 17183667 10.1371/journal.pone.0000039 PMC1762398

[14] JISC. Supporting education in Virtual Worlds with virtual learning environments. Jisc, 2011 (available at http://www.jisc.ac.uk/whatwedo/programmes/elearning/ltig/vwvle.aspx).

[15] Jarvis P Holford J Griffin C Experiential learning. In The Theory and Practice of Learning: 53–67 Routledge, 2007

[16] Kneebone R Evaluating clinical simulations for learning procedural skills: a theory-based approach. Acad Med 2005; 80: 549–53 15917357 10.1097/00001888-200506000-00006

[17] Laurillard D Rethinking University Teaching: A Conversational Framework for the Effective use of Learning Technologies (3rd edn). Routledge Falmer, 2002

[18] Lee MKO Chung CMK Chen Z Acceptance of internet-based learning medium: the role of extrinsic and intrinsic motivation. Inform Manag 2005; 42: 1095–104

[19] Bonanno P Kommers MPA Exploring the influence of gender and gaming competence on attitudes towards using instructional games. Br J Educ Technol 2008; 39: 97–109

[20] Enochsson L Isaksson B Tour R Kjellin A Hedman L Wredmark T Visuospatial skills and computer game experience influence the performance of virtual endoscopy. J Gastrointest Surg 2004; 8: 876–82 15531242 10.1016/j.gassur.2004.06.015

[21] Van de Ven AH Delbecq AL Nominal groups as a research instrument for exploratory health studies. Am J Publ Health Nations Health 1972; 62: 337–42 10.2105/ajph.62.3.337 PMC1530096 5011164

[22] Braun V Clarke V Using thematic analysis in psychology. Qual Res Psychol 2006; 3: 77–101

[23] Conole G de Laat M Dillon T Darby J Student experiences of technologies. Jisc, 2006 (available at http://www.jisc.ac.uk/publications/reports/2006/lxpfinalreport.aspx).

[24] Ellgring H Nonverbal expression of psychological states in psychiatric patients. Eur Arch Psychiatry Neurol Sci 1986; 236: 31–4 3743583 10.1007/BF00641055

[25] Grober E Hamstra S Wanzel K Reznick R Matsumoto E Sidhu R The educational impact of bench model fidelity on the acquisition of technical skill: the use of clinically relevant outcome measures. Ann Surg 2004; 240: 374–81 15273564 10.1097/01.sla.0000133346.07434.30 PMC1356416

[26] Moss F McManus IC The anxieties of new clinical students. Med Educ 1992; 26: 17–20 1538650 10.1111/j.1365-2923.1992.tb00116.x

[27] Toro Troconis M Meeran K Higham J Mellstrom U Partridge M Design and delivery of game-based learning for Virtual Patients in Second Life: initial findings. In Researching Learning in Virtual Worlds (eds Peachey A Gillen J Livingstone D Smith-Robbins S): 111–38 Springer, 2010

[28] Prensky M Digital natives, digital immigrants. On Horizon 2001; 9: 1–6

